# Verbal declarative memory impairments in specific language impairment are related to working memory deficits

**DOI:** 10.1016/j.bandl.2015.01.008

**Published:** 2015-03

**Authors:** Jarrad A.G. Lum, Michael T. Ullman, Gina Conti-Ramsden

**Affiliations:** a Cognitive Neuroscience Unit, School of Psychology, Deakin University, Australia; b Department of Neuroscience, Georgetown University, United States; c School of Psychological Sciences, The University of Manchester, United Kingdom

**Keywords:** Specific language impairment, List learning, Declarative memory, Verbal learning, Memory, Working memory

## Abstract

This study examined verbal declarative memory functioning in SLI and its relationship to working memory. Encoding, recall, and recognition of verbal information was examined in children with SLI who had below average working memory (SLI_Low WM_), children with SLI who had average working memory (SLI_Avg. WM_) and, a group of non-language impaired children with average working memory (TD_Avg. WM_). The SLI_Low WM_ group was significantly worse than both the SLI_Avg. WM_ and TD_Avg. WM_ groups at encoding verbal information and at retrieving verbal information following a delay. In contrast, the SLI_Avg. WM_ group showed no verbal declarative memory deficits. The study demonstrates that verbal declarative memory deficits in SLI only occur when verbal working memory is impaired. Thus SLI declarative memory is largely intact and deficits are likely to be related to working memory impairments.

## Highlights

• Declarative memory was assessed in children with and without language impairment.
• A subgroup of language impaired children without working memory problems did not have declarative memory impairments.
• Verbal declarative memory impairments are likely to be due to poor working memory.
• Declarative memory deficits are not universal nor a core impairment in children with language impairments.

## 1 Introduction

Children with specific language impairment (SLI) have deficits in the production and comprehension of language that occur in the absence of sensory problems and intellectual impairments (American Psychiatric Association, 2013; Bishop, 1997; Leonard, 2000; World Health Organization, 1993). Different memory systems may play a role in the aetiology of SLI. Evidence has been presented showing poor working memory and procedural memory functioning is related to the language deficits in SLI (Estes, Evans, & Else-Quest, 2007; Gathercole & Baddeley, 1990; Lum, Conti-Ramsden, Morgan, & Ullman, 2013; Montgomery, 2003; Ullman & Pierpont, 2005). However, not all memory systems have been proposed to be impaired in this group. One suggestion is that declarative memory, unlike working and procedural memory, remains relatively normal in SLI, and moreover plays an important compensatory role (Lum & Conti-Ramsden, 2013; Ullman & Pierpont, 2005; Ullman & Pullman, 2015). In this investigation we examine declarative memory in SLI, and its relationship to working memory.

### 1.1 The declarative memory system

The declarative memory system encodes (or learns), stores, consolidates as well as retrieves knowledge for personal experiences (episodic memory), general knowledge about the world (semantic memory), and knowledge of words (Cabeza & Moscovitch, 2013; Eichenbaum, Sauvage, Fortin, Komorowski, & Lipton, 2012; Henke, 2010; Squire & Wixted, 2011; Ullman, 2004). Encoding knowledge or information into the system can be fast (Gluck, Meeter, & Myers, 2003). In some cases a single exposure to information or an event is sufficient for a memory to be created, stored and then retrieved after an extended period of time (Rutishauser, Mamelak, & Schuman, 2006). However, stored information is less likely to be forgotten if it can be repeatedly re-encoded from the environment and/or re-activated within the declarative memory system via consolidation processes (Alvarez & Squire, 1994; Inostroza & Born, 2013).

Much is known about the neural substrates of the declarative memory system (Squire, Stark, & Clark, 2004). During encoding the hippocampus binds different pieces of information to create a single memory trace (Eichenbaum, 2004; Mayes, Montaldi, & Migo, 2007; Squire, 1992). Evidence from clinical populations and neuroimaging of neurologically intact adults has shown structures within the medial temporal lobe are also necessary for recall and recognition of information (Gleissner, Helmstaedter, Schramm, & Elger, 2002; Haist, Shimamura, & Squire, 1992; Jones-Gotman et al., 1997; Knowlton & Squire, 1995; Lepage, Habib, & Tulving, 1998; Moser & Moser, 1998; Spaniol et al., 2009; Squire, 1992).

Regions within the prefrontal cortex also play a role in encoding and retrieving information from declarative memory (Blumenfeld & Ranganath, 2007; Dolan & Fletcher, 1997; Fletcher, Shallice, & Dolan, 1998; Fletcher, Shallice, Frith, Frackowiak, & Dolan, 1998; Nyberg, Cabeza, & Tulving, 1996; Sandrini, Censor, Mishoe, & Cohen, 2013; Simons & Spiers, 2003). The dorsolateral prefrontal cortex (DLPFC) has been found to be active when multiple items are to be encoded into declarative memory. Under these conditions the DLPFC re-organises items together on the basis of similar semantic or perceptual features (Blumenfeld, Parks, Yonelinas, & Ranganath, 2011; Blumenfeld & Ranganath, 2006; Long, Öztekin, & Badre, 2010). Presumably, this permits more information to be encoded because fewer neural resources are required to represent the incoming information. The ventrolateral prefrontal cortex (VLPFC) aids encoding by directing attention to salient features of information and disengaging attention from irrelevant information (Badre & Wagner, 2007; Raye, Johnson, Mitchell, Reeder, & Greene, 2002). With respect to retrieval, the DLPFC plays a role in monitoring information retrieved from declarative memory (Badre & Wagner, 2007; Henson, Shallice, & Dolan, 1999; McLaughlin, Moore, Fulwiler, Bhadelia, & Gansler, 2009; Rugg, Fletcher, Chua, & Dolan, 1999). Evidence has been presented suggesting that VLPFC is involved in selecting cues that are used to retrieve information from declarative memory (Badre, Poldrack, Paré-Blagoev, Insler, & Wagner, 2005; Dobbins, Foley, Schacter, & Wagner, 2002).

The prefrontal regions that support the encoding and retrieval of information from declarative memory also support processes associated with working memory (WM; Blumenfeld & Ranganath, 2006, 2007; Fletcher & Henson, 2001; Haxby, Petit, Ungerleider, & Courtney, 2000; Ranganath, Johnson, & D’Esposito, 2003). Working memory is involved in the short-term storage and manipulation or processing of information (Baddeley, 2003; Cowan, 1999). Prefrontal regions have been shown to subserve the working memory functions that involve the manipulation or processing of information (D’Esposito et al., 1995). For instance, the DLPFC is active when information in working memory is manipulated (D’Esposito, Postle, Ballard, & Lease, 1999). The VLPFC directs attention to information processed in working memory and/or away from distracting information (Wolf, Vasic, & Walter, 2006).

The processing and manipulation operations undertaken by working memory appear to play a role in declarative memory (Becker & Lim, 2003; Simons & Spiers, 2003; Stebbins, Gabrieli, Masciari, Monti, & Goetz, 1999; Wagner, 1999). One source of evidence to support this claim has been forwarded from fMRI studies with healthy adults. In one study Blumenfeld and Ranganath (2006) asked participants to re-order a list of words on the basis of their physical characteristics (e.g., weight). Participants completed this task whilst in an MRI scanner. After scanning was completed participants were given a surprise recognition task. Their goal was to recognise words they had re-ordered in the scanner from distractor items. A key result to emerge was that the DLPFC activation associated with re-ordering the words, predicted success on the recognition task. In interpreting these results it was suggested that working memory supports encoding of information into declarative memory by re-organising or chunking information prior to being encoded into the hippocampus. In another study Cabeza, Dolcos, Graham, and Nyberg (2002) found DLPFC was activated when participants engaged in a recognition task and whilst temporarily storing a word. These data might suggest that working memory serves as a temporary hold to monitor information retrieved from declarative memory.

There is behavioural data consistent with the proposal that common processes may support working memory and declarative memory. Research into the latent structure of the Wechsler Memory Scale-III (Wechsler, 1997), which is a standardised memory test for adolescents and adults, indicates the working memory construct correlates with the declarative memory construct. Millis, Malina, Bowers, and Ricker (1999) found the correlation between working memory and declarative memory for verbal information (which includes the encoding and retrieval of information) to be .65. The correlation between working memory and declarative memory for visual information was found to be .49. Using Cohen’s (1988) convention the magnitude of the correlation between working memory and declarative memory can be considered to be ‘large’.

During childhood there also appears to be an association between working memory and the encoding and retrieval of information from declarative memory. The association between these two memory system has been examined to investigate the validity of the Children’s Memory Scale (Cohen, 1997a). The CMS is a standardised test for assessing memory functioning in children and adolescents. The subtests that comprise this instrument are similar to the WMS-III (Wechsler, 1997). Using data from the standardisation sample, the correlation between a composite scale that measures verbal working memory and a scale that measures encoding and retrieval of verbal information from declarative memory, is reported to be .41 (Cohen, 1997b). Thus at the behavioural level the association between working memory and declarative memory appears to be present from childhood to adulthood.

### 1.2 Declarative memory in specific language impairment

The ability to encode and retrieve information via declarative memory in SLI has been examined for verbal and non-verbal information. Evidence suggests that declarative memory for non-verbal information such as for unknown faces or abstract visual stimulus remains largely normal in SLI, as tested with a variety of paradigms probing encoding, recall, and recognition (e.g., Baird, Dworzynski, Slonims, & Simonoff, 2010; Bavin, Wilson, Maruff, & Sleeman, 2005; Lum, Gelgec, & Conti-Ramsden, 2010; for a review see Lum & Conti-Ramsden, 2013; Riccio, Cash, & Cohen, 2007). The status of verbal declarative memory in SLI is less clear, with some studies report impairments as compared to typically developing individuals (Dewey & Wall, 1997; McGregor et al., 2013; Nichols et al., 2004), and others finding no evidence of such deficits (Baird et al., 2010; Records, Tomblin, & Buckwalter, 1995; Shear, Tallal, & Delis, 1992).

Verbal declarative memory in SLI and other disorders has been commonly assessed using word list-learning tasks (Baron, 2004; Lezak, 2004). These tasks typically consist of encoding (learning) and retrieval (recall, recognition) phases. During the encoding phase participants are auditorily presented with a list of words. The list is presented three, four or five times, depending on the task. After each trial (i.e., after each presentation of the list), the participant is asked to recall all the words. Performance on this part of the task is taken to index encoding abilities.

The performance of children with SLI on list learning tasks has been widely investigated (for a review see Lum & Conti-Ramsden, 2013). A well-replicated finding is that participants with SLI perform worse than age-matched peers during the encoding phase (Baird et al., 2010; Dewey & Wall, 1997; Duinmeijer, de Jong, & Scheper, 2012; Lum & Bleses, 2012; Lum, Conti-Ramsden, Page, & Ullman, 2012; Nichols et al., 2004; Records et al., 1995; Riccio et al., 2007; Shear et al., 1992). That is, even after repeated exposures to a word list, individuals with SLI recall fewer words from the list when compared to typically developing (TD) peers.

It is not clear whether SLI is associated with a retrieval deficit from declarative memory. With respect to immediate recall, although some studies have found deficits (Lum & Bleses, 2012; Lum, Conti-Ramsden, Page, et al., 2012; Nichols et al., 2004), others have not (Baird et al., 2010; Records et al., 1995; Shear et al., 1992). Similarly, on measures of delayed recall reports of impairment in SLI have been observed (Lum, Conti-Ramsden, Page, et al., 2012; Nichols et al., 2004; Shear et al., 1992), but this result has not always been replicated (Baird et al., 2010; Riccio et al., 2007). However, studies that have not observed poor recall in SLI have controlled for performance on the encoding phase of the learning task (Baird et al., 2010; Records et al., 1995). This finding raises the possibility that the difficulty individuals with SLI have with retrieval, could be secondary to an encoding deficit (McGregor et al., 2013; Nichols et al., 2004). That is, since fewer items are learnt, there is less to be recalled.

The evidence suggests poorer verbal recognition in SLI. On this part of the list learning task participants with SLI have consistently been found to perform significantly poorer than age matched peers (Lum, Conti-Ramsden, Page, et al., 2012; Nichols et al., 2004; Riccio et al., 2007; Shear et al., 1992). However it needs to be noted that only delayed, not immediate, recognition, has been tested in SLI. Also studies have not usually adjusted recognition scores to take into account the number of items learnt during the encoding phase (Lum, Conti-Ramsden, Page, et al., 2012; Nichols et al., 2004; Riccio et al., 2007; Shear et al., 1992). In accounting for these results it could be that recognition problems in this group are also secondary to encoding deficits (McGregor et al., 2013).

Another possibility is that the working memory problems typically found in children with SLI may contribute to the difficulties this group have with encoding and retrieving information from declarative memory. The evidence suggests encoding and retrieval of information from declarative memory are likely to be supported by working memory. Also there are common brain networks supporting processes undertaken by both memory systems (Blumenfeld & Ranganath, 2007; Dolan & Fletcher, 1997; Sandrini et al., 2013; Simons & Spiers, 2003). As noted earlier, during encoding, working memory appears to play a role in organising information (Blumenfeld & Ranganath, 2006). Also working memory may also monitor information that has been retrieved from declarative memory (Cabeza et al., 2002).

The co-occurrence of working memory and declarative memory problems in SLI may be due to prefrontal dysfunction. Consistent with this view, in a fMRI study Ellis Weismer, Plante, Jones, and Tomblin (2005) found lower DLPFC activation in SLI whilst engaged in a verbal working memory task. In SLI, approximately three quarters (about 75–80%) of children appear to have poor working memory (Alloway, Rajendran, & Archibald, 2009; Ellis Weismer, Evans, & Hesketh, 1999). However, there is a sizeable proportion (20–25%) of children with SLI who appear to have normal or at least average working memory abilities. This situation affords a ‘natural experiment’ whereby one can examine the potential relationship between working and declarative memory in SLI. In this study we tested a claim from the Procedural Deficit Hypothesis of SLI (Ullman & Pierpont, 2005). According to this model the language problems in SLI arise from dysfunction of the basal ganglia that leads to a procedural memory impairment in this group. However, the model maintains that the medial temporal lobes, which support the declarative memory system, are intact in this population. If this claim is correct, it would be expected that children with SLI who do not have verbal working memory impairments should perform at comparable levels as control on a declarative memory task.

### 1.3 The current study

In this study we tested whether verbal declarative memory deficits in SLI are associated with verbal working memory deficits. The investigation uses a three-group comparison design. A group of children with SLI with below average (impaired) working memory (hereafter referred to as SLI_Low WM_). A group of children with SLI with average working memory (SLI_Avg. WM_) and a third group comprising typically developing children without language impairments, and with average working memory (TD_Avg. WM_). All children in the study were presented with a word list-learning task that measured encoding, recall, and recognition for verbal information. If verbal declarative memory difficulties in SLI are related to working memory problems in the disorder, then difficulties with encoding and retrieving verbal information should only be observed in the SLI group with poor working memory and not in the other SLI or TD comparison groups.

## 2 Method

### 2.1 Participants

Participants in this study were drawn from a larger sample comprising typically developing children (*n* = 57) and those with SLI (*n* = 58). All children with SLI were receiving in-school support for a language problem. None of the children had previously been diagnosed with hearing, visual or medical conditions as determined by parental questionnaire (for details see Lum, Conti-Ramsden, & Ullman, 2012). All participants were recruited from the North East of England. The children with SLI and TD children were recruited from the same schools. The data from the word list-learning task presented in this study has not previously been reported.

Three groups of children were selected on the basis of their verbal working memory and language skills. Verbal working memory was assessed using the Central Executive Component Score from the Working Memory Test Battery for Children (WMTB-C; Pickering & Gathercole, 2001a). The Central Executive Component Score and Phonological Loop Component Score are standardised to a mean of 100 and standard deviation of 15. Children’s language skills were measured using the Clinical Evaluation of Language Fundamentals-4th Edition, UK Standardisation (CELF-4^UK^; Semel, Wiig, & Secord, 2003a). The CELF-4^UK^ yields three composite scores. Children’s ability to understand and produce language is measured by the Receptive Language Index (RLI) and the Expressive Language Index (ELI), respectively. Children’s overall language ability is measured by the Core Language Score (CLS). The CLS is a composite score that is obtained by summing the RLI and ELI. The RLI, ELI and CLS are standardised to a mean of 100 and standard deviation of 15. A detailed description of the WMTB-C and CELF-4^UK^ is presented in Section 2.2.

Two groups comprised children with SLI and a third, typically developing children. One of the SLI groups (*n* = 19; 26.3% female) consisted of children with below average or impaired verbal working memory (SLI_Low WM_) abilities in addition to impaired language. The children in the other SLI group (*n* = 16; 37.5% female) were identified to have impaired language but were found to have at least average verbal working memory (SLI_Avg. WM_) abilities. The children in the typically developing group (TD_Avg. WM_) had language and verbal working memory abilities in the average range (*n* = 17, 29.4% female). All children in the study had non-verbal intelligence abilities within the normal range. Non-verbal intelligence was measured using the Wechsler Abbreviated Scale of Intelligence (WASI; Wechsler, 1999a). A description of this test is also presented in the Section 2.2.

The children in the SLI_Low WM_ and SLI_Avg. WM_ groups were matched on the basis of general language abilities, but differed with respect to verbal working memory. Children in the SLI_Avg. WM_ and TD_Avg. WM_ were matched on the basis of verbal working memory abilities, but differed in relation to language skills. A detailed description of method used to assign and match participants is presented in Sections 2.2.4 and 2.2. Summary statistics describing the age and scores from the CELF-4^UK^ (language), WASI (PIQ) and WMTB-C for each group are presented in Table 1. Also presented in Table 1 are children’s Phonological Loop Component Score from the WMTB-C. This measures how well children temporarily store verbal information, without manipulation or processing (details of the measure are presented in Section 2.2).

Differences between groups for the variables presented in Table 1 were evaluated using ANOVA. No significant differences were found with respect to age or PIQ. In relation to the language measures, both SLI groups obtained significantly lower CLS, RLI and ELI scores compared to the TD group. There were no significant differences between the SLI_Low WM_ and SLI_Avg. WM_ groups on the language measures. Finally, the Central Executive Component score for the SLI_Low WM_ was significantly lower than the SLI_Avg. WM_ and TD_Avg. WM_ groups. Interestingly, both the SLI groups were found to have poorer phonological short term memory that the TD group. Thus overall, the two SLI groups had comparable phonological short-term memory. However, only the SLI_Low WM_ group had poorer verbal working memory. An additional analysis also showed there were no significant differences in the number of females in each group (*χ*^2^ (2) = 0.5, *p* = .779).

### 2.2 Materials

#### 2.2.1 Language assessment

Language skills were assessed using the Clinical Evaluation of Language Fundamentals-4th Edition, UK Standardisation (CELF-4^UK^; Semel et al., 2003a). The CELF-4^UK^ is a standardised language test suitable for children and adolescents aged between 5 and 16 years. All children with SLI in the sample obtained a CLS of 85 or less (i.e., their language skills were more than 1 *SD* below the normative mean). This criterion has shown to have a high level of diagnostic accuracy in a UK sample (Sensitivity = 1.00, Specificity = 0.82; Semel, Wiig, & Secord, 2003b). Children in the SLI_Low WM_ and SLI_Avg. WM_ groups were selected to have the same CLS scores (±5 points). All typically developing children obtained a CLS that was between 94 and 111 (i.e., scored within the average range). The average reliability for the CLS, ELI and RLI is reported to be .96, .90 and .93 respectively (Semel et al., 2003b).

#### 2.2.2 Non-verbal intelligence

Non-verbal intelligence was measured using the Performance IQ (PIQ) from the Wechsler Abbreviated Scale of Intelligence (WASI; Wechsler, 1999a). All children participating in the study had PIQ between 90 and 115 (i.e., in the average range). The average reliability for the PIQ is .94 (Wechsler, 1999b).

#### 2.2.3 Working memory assessment

Children’s verbal working memory was assessed using the Working Memory Test Battery for Children (WMTB-C; Pickering & Gathercole, 2001a). The WMTB-C is a standardised test for assessing working memory and short-term memory. The test is standardised for children aged between 5 and 15 years. Verbal working memory is measured by the ‘Central Executive Component Score’. This composite score is obtained by summing performance from three verbal working memory subtests: Backward Digit Recall, Counting Recall and Listening Recall. The reliabilities for the Backward Digit Recall, Counting Recall and Listening Recall has been reported to be .83, .71 and .73 respectively (Gathercole, Alloway, Willis, & Adams, 2006). All three subtests require the examinee to temporarily store and then process or manipulate verbal information. Thus the Central Executive Component Score can be considered to measure how well children can complete the combined operations necessary to support the short-term storage and processing/manipulation of verbal information.

The Backward Digit Recall subtest requires children to repeat an increasingly longer string of digits in reverse order. On the Counting Recall subtest children first count an array of randomly placed dots. After the dots have been counted children are asked to recall how many dots were shown. The subtest increases in difficulty as children are asked to count and remember an increasing number of dot arrays, prior to recall. The Listening Recall subtest requires children to listen to an increasing number of sentences (e.g., ‘dogs have four legs’). After each sentence has been presented children are asked whether it is ‘true’ or ‘false’. After all sentences have been presented children are asked to recall all sentence final words. The Central Executive Component Score is standardised to a mean of 100 and standard deviation of 15.

The children’s ability to temporarily store verbal information was assessed using the Phonological Loop Component Score. The Phonological Loop Component Score is obtained by summing performance from four subtests: Digit Recall, Word List Matching, Word List Recall and Nonword List Recall. Only the test–retest reliabilities have been reported for these subtests. For Digit Recall the test–retest reliability has been reported to be .81, for Word List Matching .45, for Word List Recall .80 and Nonword List Recall .68 (Pickering & Gathercole, 2001b). Common to all subtests is that children need to temporarily store an increasing amount of verbal information.

On the Digit Recall subtest children are asked to repeat an increasingly longer string of numbers. On the Word List Recall children repeat back a word list that increases in number. The Nonword List Recall is similar except participants repeat lists of nonwords. On the Word List Matching subtest children are presented with a sequence of words twice. Children’s task is to determine whether the order words were presented was the same on each trial.

#### 2.2.4 Matching criteria

A child was considered to have a verbal working memory impairment if s/he obtained a Central Executive Component Score that was 85 or less (Gathercole & Alloway, 2006). That is, −1 *SD* or more from the normative mean. Children with SLI were selected for the SLI_Low. WM_ group if they obtained a Central Executive Component Score that was 85 or less. Children in the SLI_Avg. WM_ and TD_Avg. WM_ groups obtained a Central Executive Component Score that was 90 or higher. In addition, children in the SLI_Avg. WM_ and TD_Avg. WM_ groups were individually matched using the Central Executive Component Score (±5 points). The Central Executive Component Score was used to create the groups because processes tapped by this composite have been linked to the operations subserved by the DLPFC and VLPFC (D’Esposito et al., 1999; Ranganath, Johnson, & D’Esposito, 2003). It is these regions that may contribute to encoding and also retrieval processes from declarative memory (Blumenfeld & Ranganath, 2007; Dolan & Fletcher, 1997; Sandrini et al., 2013; Simons & Spiers, 2003).

#### 2.2.5 List learning task

The Word List subtest from the Children’s Memory Scale (CMS; Cohen, 1997a) was used to measure the encoding and retrieval of verbal information from declarative memory (CMS; Cohen, 1997a). Performance on list learning tasks has shown to be impaired following damage to the left medial temporal lobe (Gleissner et al., 2002; Jambaqué, Dellatolas, Dulac, Ponsot, & Signoret, 1993; Jones-Gotman et al., 1997).

During the encoding phase of the task children are auditorily presented with a 14-item word list four times. The words in the list are not designed to be semantically related (the words in the list are: car, forest, dog, night, paper, hand, metal, rock, line, window, farmer, watch, sound, bank). After the first presentation of the list children are asked to recall as many words as possible. The word list is then presented three more times. On the second to fourth presentations the test administrator only presents words that the child omitted. The child is asked to recall as many words as possible, including those recalled in a previous trial. The reliability for this part of the task has been reported to be .86 (Cohen, 1997b).

The retrieval of verbal information was assessed in immediate and delayed recall conditions, and in a delayed recognition condition. The retrieval conditions followed the four encoding trials and a subsequent 14-item distractor list, which children also had to recall. The immediate recall phase was presented immediately after the distractor list. In this condition children were asked to recall words from the initial word list. The delayed recall and recognition conditions were administered after a delay of about 15 min, during this time other subtests from CMS were administered (for a summary of these findings see Lum, Conti-Ramsden, Page, et al., 2012). In the delayed recall phase children were asked to recall as many words from the initial word list as possible. For the delayed recognition component, children were auditorily presented with 42 words and asked to indicate which ones were presented earlier. The items from this part of the task comprise 21 targets and 21 foils of comparable syllabic length. The 21 targets comprised all items from the word list with seven of the 14 items presented twice. Similarly, seven of the foils were presented twice. All items were analysed. The 14 foils were: nose, door, van, glue, circle, desert, iron, stone, pig, dark, tank, music, painter, clock. The reliability for the delayed recall and recognition is .72 and .77 respectively (Cohen, 1997b).

A measure of verbal encoding on the task was obtained by summing the total number of correct words recalled on each trial. The maximum score that could be obtained on each trial was 14. Performance on the immediate and delayed recall phase was measured using two approaches. In the first approach the total number of items recalled was computed as a proportion of the total number of words in the list (i.e., *n* = 14). These scores are hereafter referred to as ‘Unadjusted Recall Scores’. However, as noted in the Introduction children with SLI may encode fewer words and a recall score based on the entire word list may underestimate recall ability. Subsequently a second approach to measure immediate and delayed recall was computed using a method that took into account which words were successfully encoded. Specifically a word from the list was considered to have been encoded if it was recalled at least twice during the encoding phase (nb. increasing the threshold to three items recalled resulted in floor effects). These scores are hereafter referred to as ‘Adjusted Recall Scores’. The requirement that the word be recalled more than once was used because correct recall on a single trial may reflect short-term memory. The number of words recalled in immediate and delayed conditions was calculated based on successfully encoded words only. This adjustment allowed differences between groups on retrieval measures to be investigated whilst controlling for encoding differences.

A measure of delayed recognition was obtained using the *d*-prime statistic from signal detection theory. In studies of recognition memory this statistic is preferred because it corrects for potential response bias (Green & Swets, 1966), such as responding ‘Yes’ to every item. This statistic is also commonly used in standardised tests of recognition memory in list learning tasks (Delis, 1994; Schmidt, 1996; Sheslow & Adams, 1990). The *d*-prime statistic is computed from the number of ‘Hits’ and ‘False Alarms’ (Green & Swets, 1966). In the context of the List Learning subtest, ‘Hits’ describe trials during the recognition phase when a word was correctly identified as belonging to a word presented during the encoding phase. ‘False Alarms’ are responses where a foil was incorrectly identified as belonging to the word list. *d*-Prime values greater than zero indicate increasing ability to discriminate between targets and foils.

For each participant an ‘Unadjusted’ and ‘Adjusted’ *d*-prime value was computed using an approach similar to the methods used to quantify recall. The ‘Unadjusted’ *d*-prime value was computed using all items from the encoding phase irrespective of whether the word was considered to have been encoded. The ‘Adjusted’ *d*-prime value took into account performance on the encoding phase. Specifically, a ‘Hit’ was computed only for words that were identified as having been learned during the encoding phase that is, that were correctly recalled at least twice during that phase.

### 2.3 Procedure

Children were individually presented with the language, working memory and list-learning tasks in a quiet room at their respective school. The same research assistant presented all tests to the children and was done according to test guidelines. Presentation of the tests was randomised. These tasks were presented as part of a larger assessment battery examining language and memory. Ethical approval for the study was obtained from The University of Manchester, and informed written consent was gained from the children’s parents or legal guardians.

## 3 Results

### 3.1 Encoding

**Fig. 1:**
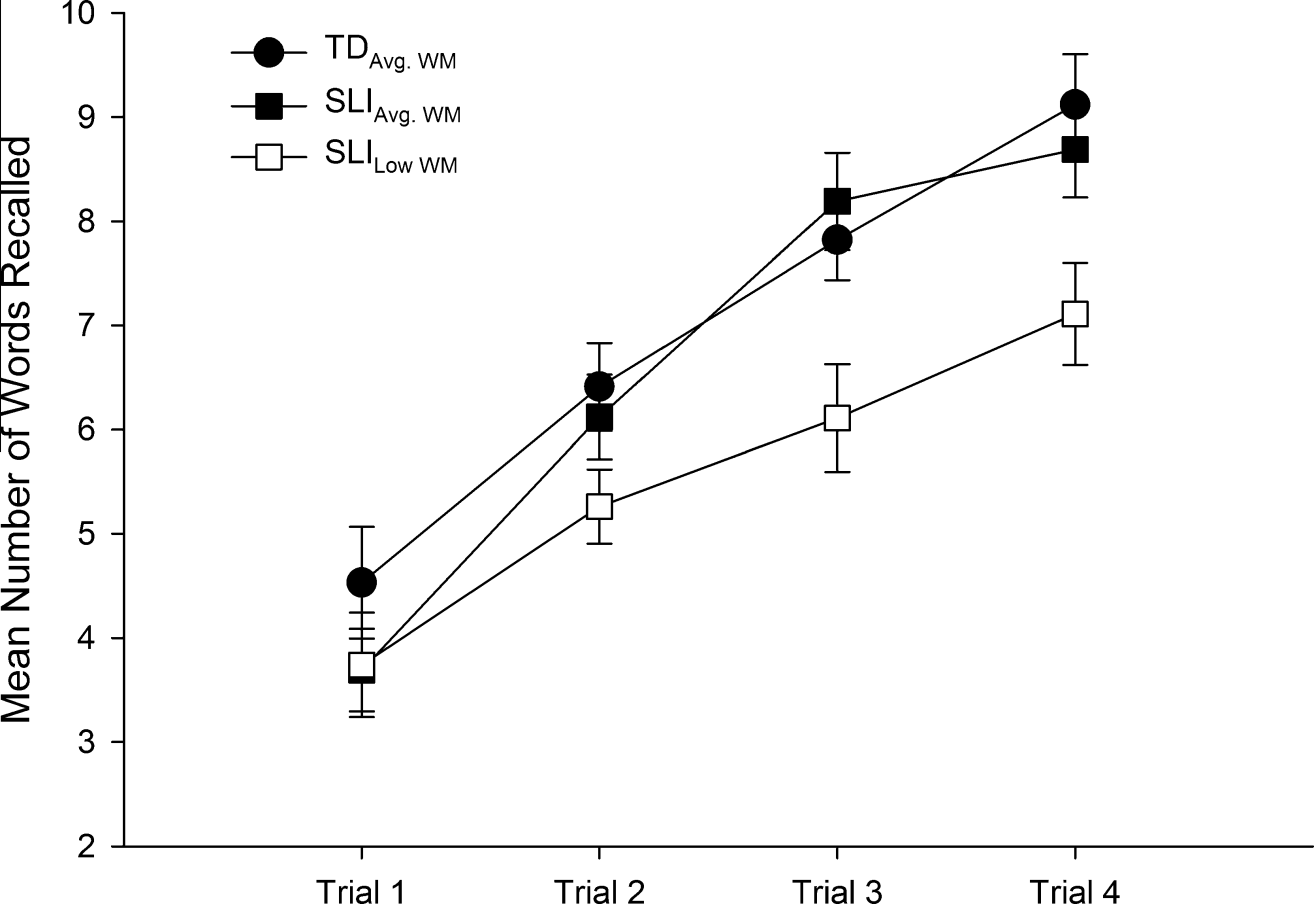
Mean number of words recalled during the encoding phase presented by group. The maximum number of words that could be recalled on a single trial is 14. Error bars show standard error.

The first set of analyses investigated group differences on the encoding phase. Data from this part of the task are summarised in Table 2 and for illustrative purposes presented in Fig. 1. These data were submitted to a 3 (Group: SLI_Low WM_, SLI_Avg. WM_, TD_Avg. WM_) × 4 (Trial: Trial 1, Trial 2, Trial 3, Trial 4) Mixed Design Factorial ANOVA. This analysis revealed a significant main effect for Group (*F*(2, 49) = 4.406, *p* = .017, *partial η*^2^ = .152) and Trial (*F*(3, 147) = 102.174, *p* < .001, *partial η*^2^ = .676). A significant Group × Trial interaction (*F*(6, 147) = 2.288, *p* = .038, *partial η*^2^ = .085) was also observed.

Following up on the interaction, we performed one-way ANOVAs to investigate group differences at each encoding trial. For all such post hoc tests, *p*-values were adjusted for multiple comparisons using the Holm’s procedure (Holm, 1979). These analyses revealed no group differences on Trial 1 (*p* = .402) or Trial 2 (*p* = .200), while significant group differences emerged on Trial 3 (*p* = .030) and Trial 4 (*p* = .020). Post hoc *t*-tests indicated that on Trials 3 and 4, the SLI_Low WM_ scores were significantly lower than both the SLI_Avg. WM_ and TD_Avg. WM_ groups (Trial 3: SLI_Low WM_ vs. SLI_Avg. WM,_ *p* = .022; SLI_Low WM_ vs. TD_Avg. WM_, *p* = .009; Trial 4: SLI_Low WM_ vs. SLI_Avg. WM,_ *p* = .048; SLI_Low WM_ vs. TD_Avg. WM_, *p* = .012). In contrast, no significant differences were observed between the SLI_Avg. WM_ and TD_Avg. WM_ on Trial 3 (*p* = .593) or Trial 4 (*p* = .541).

It is evident from Fig. 1 that all groups demonstrated an increase in the number of words recalled across the encoding phase. Planned contrasts using repeated measures ANOVA undertaken separately for each group were performed to test whether there was a linear increase in words recalled from Trial 1 to Trial 4. A significant linear increase was observed for all three groups (all *p*’s < .001). Overall, these results show that all groups encoded verbal information following repeated exposure to the words; however by Trial 3, and continuing to Trial 4, the SLI_Avg. WM_ and TD_Avg. WM_ had encoded more words than the SLI_Low WM_ group.

### 3.2 Immediate and delayed recall

**Fig. 2:**
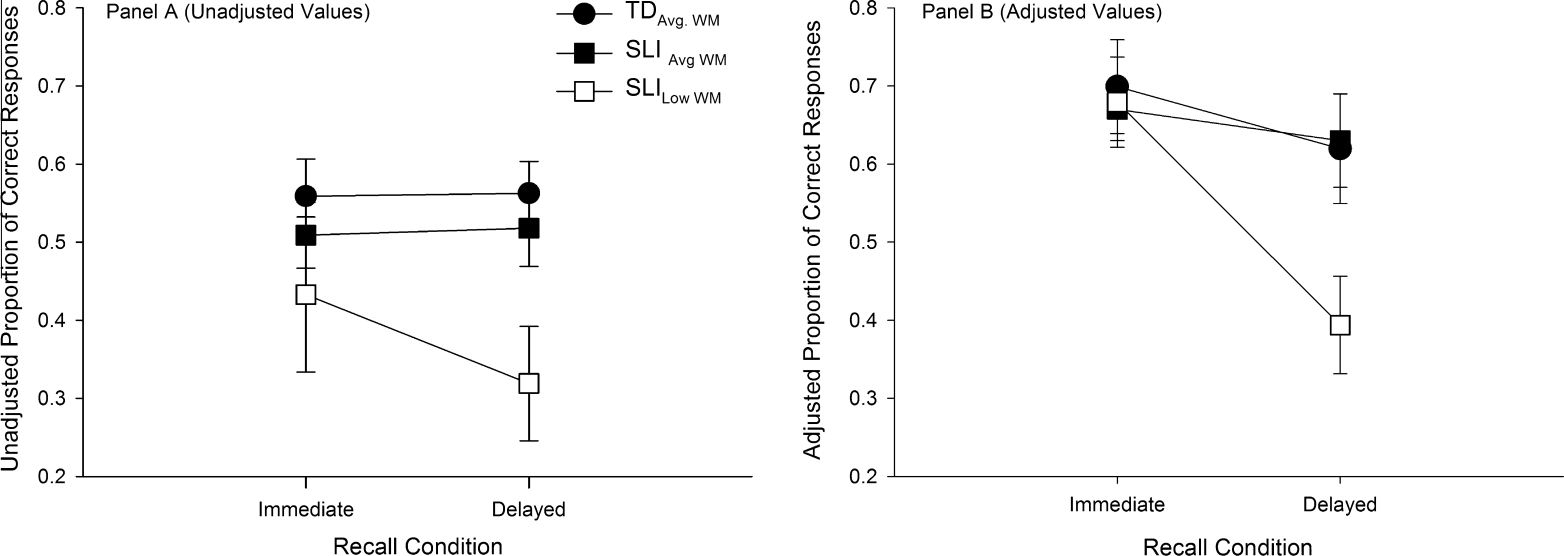
Proportions of recalled during the immediate and delayed recall condition reported by group. Panel A showed unadjusted recall scores. Panel B shows adjusted recall scores. Error bars show standard error.

The next set of analyses investigated differences between the groups on the measures of immediate and delayed recall. First, differences between groups were examined using Unadjusted Immediate and Unadjusted Delayed Recall Scores. This approach to measuring recall performance did not take into account children’s performance on the encoding phase. Both Unadjusted and Adjusted recall scores are summarised in Table 2. Also, for illustrative purposes these data are summarised in Fig. 2, Panel A.

Unadjusted Recall scores were analysed using a 3 (Group: SLI_Low WM_, SLI_Avg. WM_, TD_Avg. WM_) × 2 (Recall Condition: Immediate, Delayed) Mixed-Design Factorial ANOVA. This analysis revealed a significant main effect for Recall Condition (*F*(1, 49) = 5.940, *p* = .018, *partial η*^2^ = .108) and Group (*F*(1, 49) = 8.149, *p* = .001, *partial η*^2^ = .250). The interaction between Group and Recall was also significant (*F*(1, 49) = 4.236, *p* = .020, *partial η*^2^ = .147).

To examine the source of the interaction one-way ANOVA’s were first conducted to test for differences between the groups on the Unadjusted Immediate Recall and then Unadjusted Delayed Recall scores. There was a significant difference between the groups on the Unadjusted Immediate Recall scores (*F*(2, 49) = 3.934, *partial η*^2^ = .138). Post hoc tests revealed the TD group recalled significantly more words than the SLI_Low WM_ group (*p* < .001). The SLI_Avg. WM_ group also recalled more words than the SLI_Low WM._ However_,_ the difference fell short of statistical significance (*p* = .064). The difference between the TD group and SLI_Avg. WM_ group was not significant (*p* = .453) The results from the ANOVA examining differences between the groups on Unadjusted Delayed Recall scores also revealed a significant difference between groups (*F*(2, 49) = 10.668, *p* < .001, *partial η*^2^ = .303).

The next set of post hoc tests compared differences in Unadjusted Immediate and Delayed Recall scores within each group. No significant difference in recall between immediate and delayed conditions was observed for the TD (*p* = .402) or SLI_Avg. WM_ group (*p* = .751). However, the SLI_Low WM_ recalled significantly fewer words in the delayed recall conditions compared to the immediate recall condition (*p* = .006).

The next analyses examined the groups’ performance using Adjusted Immediate Recall and Adjusted Delayed Recall scores. As noted earlier, Adjusted Scores took into account children’s performance on the encoding phase (see Section 2). Specifically, an item was considered to have been encoded if it was recalled at least two times during the encoding phase. Using this criterion the average number of words encoded by the TD_Avg. WM_ group was 9.1 (*SD* = 2.17, Range: 6–13), for the SLI_Avg. WM_ group 8.1 (*SD* = 1.55, Range: 6–11) and for the SLI_Low WM_ group 7.39 (*SD* = 2.43, Range: 4–13). One-way ANOVA was undertaken to examine group differences in the number of words encoded. The effect of group fell short of statistical significance (*F*(2, 49) = 2.665, *p* = .080, *partial η*^2^ = .104). However, given that the effect size for this analysis was large the non-significant result likely reflects insufficient statistical power.

Adjusted Recall scores are presented in Table 2 and also in Fig. 2, Panel B. These data were analysed using a 3 (Group: SLI_Low WM_, SLI_Avg. WM_, TD_Avg. WM_) × 2 (Recall Condition: Immediate, Delayed) Mixed-Design Factorial ANOVA. This analysis revealed a significant main effect for Recall Condition (*F*(1, 49) = 12.422, *p* = .001, *partial η*^2^ = .202), but not for Group (*F*(2, 49) = 2.135, *p* = .129, *partial η*^2^ = .080), while the interaction between Group and Recall Condition was significant (*F*(2, 49) = 3.952, *p* = .026, *partial η*^2^ = .139). Following up on the interaction, one-way ANOVAs revealed no significant differences between groups on the immediate recall condition (*p* = .937). However, a significant group difference on delayed recall was observed (*p* = .026). Post hoc *t*-tests showed that the SLI_Low WM_ group recalled significantly fewer words in the delayed recall condition compared to both the SLI_Avg. WM_ (*p* = .033) and TD_Avg. WM_ groups (*p* = .031). In contrast, the difference between the SLI_Avg. WM_ and TD_Avg. WM_ groups (*p* = .925) was not significant.

#### 3.2.1 Delayed recognition

**Fig. 3:**
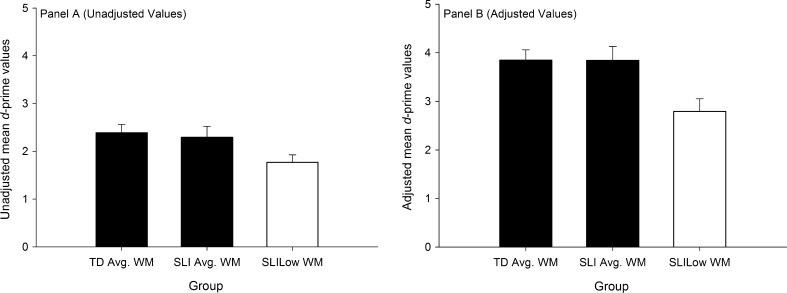
Mean *d*-prime values for delayed recognition reported by group. Panel A shows unadjusted *d*-prime values. Panel B shows adjusted *d*-prime values. Error bars show standard error.

The final analysis tested group differences in delayed recognition. In these analyses separate one-way ANOVAs were used to investigate differences between groups for Unadjusted and Adjusted *d*-prime values. Unadjusted *d*-prime values did not take into account children’s performance on the encoding phase. Summary statistics for these values reported by group is presented in Table 2 and also in Fig. 3, Panel A. Adjusted *d*-prime values were computed only using items that were identified to have been encoded. These data are summarised in Fig. 3, Panel B.

A significant difference between groups was observed when the Unadjusted *d*-prime value was entered as the dependent variable (*F*(1, 49) = 8.795, *p* < .001, *partial η*^2^ = .300). Post hoc tests revealed that the Unadjusted *d*-prime values for the SLI_Low WM_ group were significantly lower compared to the SLI_Avg. WM_ (*p* = .003) and TD_Avg. WM_ groups (*p* < .001). The difference between the TD_Avg. WM_ and SLI_Avg. WM_ groups was not significant (*p* = .437).

A similar pattern of results was observed when using Adjusted *d*-prime values as the dependent variables. Results revealed a significant main effect for Group, *F*(2, 49) = 5.907, *p* = .005, *partial η*^2^ = .194. Post hoc *t*-tests revealed that the SLI_Low WM_ group obtained significantly lower *d*-prime values during the recognition phase than both the SLI_Avg. WM_ (*p* = .015) and TD_Avg. WM_ groups (*p* = .015). No significant differences were observed between the SLI_Avg. WM_ and TD_Avg. WM_ group (*p* = .988).

## 4 Discussion

This study investigated the role of verbal working memory in the encoding and retrieval of verbal information in SLI. The question addressed in the study was whether encoding and retrieval deficits reported in SLI for verbal declarative memory are related to verbal working memory problems (Baird et al., 2010; Dewey & Wall, 1997; Duinmeijer et al., 2012; Lum, Conti-Ramsden, Page, et al., 2012; Nichols et al., 2004; Records et al., 1995; Riccio et al., 2007; Shear et al., 1992). Results from this study provide some support for this proposal. Specifically, the SLI_Avg. WM_ group were able to encode and retrieve verbal information at a level comparable to the TD_Avg. WM_ group. Thus the declarative memory system is not impaired in children with SLI who do not have verbal working memory problems. However, in SLI poor verbal working memory does appear to negatively impact on the encoding of verbal information and retrieval of verbal information following a delay. Overall the results suggest poor declarative memory for verbal information in SLI is more closely related to working memory than language problems.

Research investigating declarative memory for verbal information in SLI has consistently observed encoding deficits in SLI (Baird et al., 2010; Dewey & Wall, 1997; Duinmeijer et al., 2012; Lum, Conti-Ramsden, Page, et al., 2012; McGregor et al., 2013; Nichols et al., 2004; Records et al., 1995; Riccio et al., 2007; Shear et al., 1992). This has led to the suggestion that SLI is associated with a verbal encoding impairment (McGregor et al., 2013; Nichols et al., 2004). The results of this study shed new light about the nature of this encoding difficulty. In the first instance, the problems appear to be related to verbal working memory and not phonological short-term memory. As reported earlier (see Table 1) no significant differences were found between the two SLI groups on the measure of phonological short-term memory. Second, problems with verbal encoding are not universal in SLI: statistically indistinguishable performance at encoding was observed between the SLI_Avg. WM_ and TD_Avg. WM_ groups. Verbal encoding problems were only present in those children with SLI with verbal working memory impairments.

It is thought that working memory might aid encoding information into declarative memory by clustering similar items on the basis of semantic or perceptual features (Blumenfeld & Ranganath, 2006). Poor verbal working memory may limit the extent these processes can be deployed or the number of items that can be re-organised. This may explain the performance of the SLI_Low WM_ group on the encoding phase. Analyses showed that the number of words recalled by this group increased linearly from Trial 1 to Trial 4. However, the overall number of words learnt was lower compared to the groups (both SLI and TD) with average working memory. Interestingly, this problem does not appear to be related to language difficulties since the SLI group without verbal working memory problems performed at levels comparable to the TD group.

In relation to retrieval, the results indicate poor verbal working memory in SLI might be negatively influencing delayed but not immediate recall. On the immediate recall phase of the list-learning task the difference between the SLI_Low WM_ and the SLI and TD groups with average working memory was not significant after performance on the encoding phase was controlled.

In SLI, poor verbal working memory may negatively impact on the delayed recall and recognition of verbal information from declarative memory. Analyses showed that the SLI_Low WM_ group was less accurate than the two average working memory groups (both SLI and TD) on the delayed retrieval conditions. These results were found even after scores were adjusted to take into account performance on the encoding phase. In the first instance problems with delayed recall do not seem to reflect a fundamental problem with recall more generally. As noted earlier, after controlling for differences in encoding the SLI_Low WM_ group were comparable to the SLI and TD groups with average working memory. One possibility is that delayed retrieval of information increases demands on working memory. In the context of a list-learning task this might occur because the amount of time between encoding and retrieval has increased and there is more irrelevant information to ignore.

The SLI_Low WM_ group also performed poorer than the groups with average working memory (both SLI and TD) on the delayed recognition trials. Note that previous research has consistently shown that recognition is poorer in SLI (Lum, Conti-Ramsden, Page, et al., 2012; Nichols et al., 2004; Riccio et al., 2007; Shear et al., 1992). However, these studies have only examined recognition memory following a delay. So it is unclear whether the processes that support recognition are intact in the SLI_Low WM_ group. Indeed, it could be that recognition memory is intact in this group, but executing these processes following a delay places more demands on verbal working memory as was suggested for delayed recall. Methodological factors may also contribute to the group differences on the recognition task. The SLI and TD groups with average working memory encoded more items on the list compared to the SLI_Low WM_ group (although the difference was not significant). As a consequence the ratio of targets to foils was smaller SLI_Low WM_ group compared to the other groups. Thus the recognition task for the children in the SLI_Low WM_ group consisted of more distracter items that needed to be ignored. This may have impacted on the SLI_Low WM_ group’s performance. These children evidenced more false alarms (see Table 2) on the recognition phase compared to the groups comprising children with average working memory. That is they accepted more foils as targets. Future research into recognition memory in SLI may benefit from using a paradigm that ensures the ratio of foils to targets is equal between individuals.

Overall, the results of this study do not suggest medial temporal lobe dysfunction in SLI. This is consistent with neurological data showing medial temporal lobes are intact in SLI (Ullman, Pullman, Lovelett, Pierpont, & Turkeltaub, submitted for publication). We suggest that if SLI was associated with medial temporal lobe dysfunction we would expect to see declarative memory deficits in SLI children with both low and average working memory skills. Such deficits were only observed in the SLI group with poor verbal working memory. However, neuroimaging functional and structural data will be required to test these claims further.

Finally, the results of the study have potential clinical implications. In SLI it seems that the encoding and retrieval of verbal information is spared to varying degrees. For children with SLI who do not have verbal working memory impairments, the encoding and retrieval of verbal information from declarative memory appears to be comparable to typically developing children. For children with SLI who have poor verbal working memory, the encoding of verbal information into declarative memory certainly occurs. However, the amount of information encoded appears to be less in comparison to TD children. Children with SLI who have poor verbal working memory can also retrieve information from declarative memory at levels comparable to TD children. However, based on the data presented in this study it seems this can only be achieved when working memory demands are reduced. Subsequently, in principal, the encoding and retrieval of verbal information via the declarative memory system might be a route to scaffold learning. Future research will be required to determine whether grammatical rules can be learnt and used via the declarative memory system. If this is possible better language outcomes for children with SLI might be achievable.

In sum, evidence suggests that declarative memory deficits are not a core impairment in SLI. Previous studies have consistently reported normal non-verbal declarative memory in the disorder. In terms of verbal declarative memory, this study has showed that when impairments are observed, this is likely to be due to deficits in working memory. Future studies should examine in more detail the nature of the relationship between verbal working memory and verbal declarative memory impairments in SLI.

## Figures and Tables

**Table 1:** Summary statistics and comparison of means for age and scores from standardised tests.

Measure	Group									Comparison of means^b^	Post hoc tests
	TD_Avg. WM_			SLI_Avg. WM_			SLI_Low WM_				
	*M*	*SD*	Range	*M*	*SD*	Range	*M*	*SD*	Range		
Age (years; months)	10; 1	0; 10	9; 0–11; 4	9; 10	0; 10	8; 7–11; 5	9; 10	0; 10	8; 10–11; 5	*p* = .395	–

*Language measures*											
CLS^a^	100.7	4.8	94–111	77.1	8.8	56–85	73.4	9.2	50–84	*p* < .001	SLI_Low WM_ = SLI_Avg. WM_ < TD_Avg. WM_
ELI^a^	100.1	5.9	90–112	74.7	11.1	49–89	74.0	10.4	55–87	*p* < .001	SLI_Low WM_ = SLI_Avg. WM_ < TD_Avg. WM_
RLI^a^	100.9	8.6	88–116	81.4	8.1	67–96	76.4	6.7	61–85	*p* < .001	SLI_Low WM_ = SLI_Avg. WM_ < TD_Avg. WM_

*Intelligence measure*											
PIQ^a^	99.2	6.7	90–114	102.4	6.6	92–115	99.8	6.4	90–110	*p* = .340	–

*Verbal work. memory measure*											
Cental exec. score^a^	101.8	8.9	90–118	100.3	9.0	90–118	69.0	8.8	55–81	*p* < .001	SLI_Low WM_ < SLI_Avg. WM_ = TD_Avg. WM_
Phono. Loop Score^a^	110.8	15.3	86–141	92.5	15.0	73–133	90.7	15.8	68–132	*p* < .001	SLI_Low WM_ = SLI_Avg. WM_ < TD_Avg. WM_

Abbreviations: CLS = Core Language Score; ELI = Expressive Language Score; RLI = Receptive Language Score; PIQ = Performance IQ; Central Executive Score.

a Scores from measure are standardised to a mean of 100 and standard deviation of 15.

b Differences between means tested using one-way ANOVA.

**Table 2:** Summary statistics showing SLI and control groups’ performance on encoding and retrieval measures.

Measure	TD_Avg. WM_			SLI_Avg. WM_			SLI_Low WM_		
	*M*	*SD*	Range	*M*	*SD*	Range	*M*	*SD*	Range
*Encoding*^a^									
Trial 1 recall	4.5	2.2	3–12	3.7	1.6	2–8	3.7	2.2	1–10
Trial 2 recall	6.4	1.7	4–11	6.1	1.6	4–11	5.3	1.6	3–8
Trial 3 recall	7.8	1.6	6–11	8.2	1.9	5–12	6.1	2.3	2–10
Trial 4 recall	9.1	2.0	6–13	8.7	1.9	6–13	7.1	2.1	4–11

*Retrieval*									
Unadjusted recall scores									
Immediate	0.56	0.20	0.29–0.93	0.51	0.17	0.14–0.86	0.43	0.15	0.14–0.71
Delayed	0.56	0.17	0.36–0.93	0.52	0.20	0.36–0.93	0.32	0.17	0.07–0.64

Adjusted recall scores^b^									
Immediate	0.70	0.24	0.11–1.00	0.67	0.17	0.29–1.00	0.68	0.25	0.00–1.00
Delayed	0.62	0.28	0.00–1.00	0.63	0.22	0.00–1.00	0.39	0.27	0.00–0.90

Unadjusted recognition scores									
*d*-Prime	2.39	0.72	1.04–3.42	2.29	0.92	1.18–4.22	1.77	0.66	0.93–3.31
Hit rate	0.75	0.19	0.24–0.95	0.74	0.18	0.43–1.00	0.64	0.17	0.24–0.95
False alarm rate	0.06	0.03	0.04–0.14	0.08	0.06	0.04–0.24	0.10	0.07	0.04–0.24

Adjusted recognition scores									
*d*-Prime	3.84	0.86	1.12–4.65	3.85	1.16	1.12–4.65	2.79	1.14	1.11–4.65
Hit rate	0.88	0.06	0.82–0.99	0.89	0.12	0.56–0.99	0.82	0.21	0.33–0.99
False alarm rate	0.04	0.05	0.01–0.24	0.04	0.07	0.01–0.24	0.10	0.10	0.01–0.31

a Maximum score on each trial is 14.

b Proportion of encoded words correctly recalled.
